# Out-of-Hospital Cardiac Arrests and Outdoor Air Pollution Exposure in Copenhagen, Denmark

**DOI:** 10.1371/journal.pone.0053684

**Published:** 2013-01-14

**Authors:** Janine Wichmann, Fredrik Folke, Christian Torp-Pedersen, Freddy Lippert, Matthias Ketzel, Thomas Ellermann, Steffen Loft

**Affiliations:** 1 Section of Environmental Health, Department of Public Health, Faculty of Health Sciences, University of Copenhagen, Copenhagen, Denmark; 2 Department of Cardiology, Gentofte Hospital, University of Copenhagen, Copenhagen, Denmark; 3 Copenhagen Emergency Medical Service, Copenhagen, Denmark; 4 Department of Environmental Sciences, Aarhus University, Roskilde, Denmark; Maastricht University Medical Center, The Netherlands

## Abstract

Cardiovascular disease is the number one cause of death globally and air pollution can be a contributing cause. Acute myocardial infarction and cardiac arrest are frequent manifestations of coronary heart disease. The objectives of the study were to investigate the association between 4 657 out-of-hospital cardiac arrests (OHCA) and hourly and daily outdoor levels of PM_10_, PM_2.5_, coarse fraction of PM (PM_10-2.5_), ultrafine particle proxies, NO_x_, NO_2_, O_3_ and CO in Copenhagen, Denmark, for the period 2000–2010. Susceptible groups by age and sex was also investigated. A case-crossover design was applied. None of the hourly lags of any of the pollutants were significantly associated with OHCA events. The strongest association with OHCA events was observed for the daily lag4 of PM_2.5_, lag3 of PM_10_, lag3 of PM_10-2.5_, lag3 of NO_x_ and lag4 of CO. An IQR increase of PM_2.5_ and PM_10_ was associated with a significant increase of 4% (95% CI: 0%; 9%) and 5% (95% CI: 1%; 9%) in OHCA events with 3 days lag, respectively. None of the other daily lags or other pollutants was significantly associated with OHCA events. Adjustment for O_3_ slightly increased the association between OHCA and PM_2.5_ and PM_10_. No susceptible groups were identified.

## Introduction

Cardiovascular disease (CVD) is the number one cause of death globally and also in developed countries, such as Denmark [1]. Acute myocardial infarction (International Classification of Diseases version 10 code (ICD10) I21-22) and cardiac arrest (ICD10 I46) are frequent manifestations of coronary heart disease. Cardiac arrest represents a significant public health problem in developed countries, accounting for 250000–300000 events yearly and out-of-hospital cardiac arrests (OHCA) have a particularly poor prognosis [2]–[6].

Evidence is increasing on the effects of short- and long-term exposure to present-day outdoor air pollution levels, especially between particulate matter (PM) and CVD (all types of CVD combined) and all-cause mortality according to the American Heart Association's scientific statement – updated in 2010 [7].

PM could in principle raise the risk of OHCA in at least three ways: by advancing atherosclerosis progression during several years of exposure, by initiating or enhancing inflammatory processes in the lung and systemically within days after exposure or by triggering ventricular dysrhythmia within hours after exposure [7]–[16]. Several cohort studies support that atherosclerosis progression and mortality is associated with long-term PM levels [7], [17]–[20], and a large number of time series and case-crossover studies support a role for increased daily PM levels causing unspecific CVD admissions and mortality [21], whereas only six case-crossover studies have addressed OHCA with inconsistent results [22]–[27]. Of these only one considered hourly exposure [25], whereas none included monitoring of ultrafine particles (UFP). Traffic is the main source of UFP and the pollutant is considered important from a toxicological point of view [12], [13]. OHCA is very well defined in time and thus excellently suited to study possible lagged effects of air pollution.

The objectives of the study were to investigate the association between hourly and daily outdoor levels of traffic-related and total PM air pollution and OHCA in Copenhagen, Denmark, for the period 2000–2010 and to investigate susceptible groups by age and sex.

## Methods

### Health data

OHCAs were identified from the Mobile Emergency Care Unit (MECU) in Copenhagen covering 600 000 inhabitants from 1994–2010 [28]. Detailed information on the Copenhagen MECU has been provided elsewhere [28], [29]. In Copenhagen, a physician-staffed MECU is always deployed whenever an OHCA is suspected – along with the standard ambulance. Only in the event of more than one or two OHCAs occurring simultaneously during night or day time, respectively, there could be a chance of missing OHCA cases, as an ambulance could arrive at an OHCA patient without activating the MECU.

Cardiac arrest was defined and registered in accordance with Utstein criteria. These international criteria are a set of guidelines for uniform reporting of cardiac arrest and were first proposed for emergency medical services in 1991 [30]. Not all patients who had OHCAs were alive at inclusion, but attained return of spontaneous circulation (ROSC) following cardiopulmonary resuscitation. Patients with obvious signs of death (i.e. trauma, rigor mortis, livores) or those where resuscitation was judged effortless (i.e. ROSC not attained) by the emergency physician at the scene were not recorded as OHCAs.

All data (including date, time and occurrence) were registered and entered in the database consecutively everyday by the physician from the MECU who treated the arrest.

The time of OHCA was defined as the time the Emergency Dispatch Centre (EDC) received an OHCA call from a bystander (112-call). Next, the MECU registered the time at arrival at the OHCA location. The MECU response time was the time difference from the 112-call to arrival of the MECU [31].

The study period covered 11 years (1 January 2000 to 31 December 2010) and was determined by the availability of air pollution data. Addresses where the OHCA occurred were retrieved and geocoded. Only cases with addresses within the Copenhagen Community (about 5 km from the urban background station) were included.

### Air pollution data

Hourly meteorological and air pollution data were measured at the Copenhagen urban background monitoring station by the Department of Environmental Sciences, Aarhus University [32]. The urban background monitoring station is located on the roof of a 20 m high building in the centre of Copenhagen about 300 m east and 50 m west of streets with typical weekday traffic flows of 26 000 and 56 000 vehicles respectively, and minimal contribution from local pollution sources in accordance with World Health Organisation (WHO) guidelines.

Air pollution data included hourly average measurements of PM_10_ and PM_2.5_ (tapered element oscillating microbalance (TEOM) (Series 1400a Ambient Particulate Monitor; Thermo Fisher Scientific Inc., Franklin, MA, USA), nitrogen dioxides (NO_x_ and NO_2_) (M 200A; Teledyne API, San Diego, USA), carbon monoxide (CO) (M 300 monitor; Teledyne API, San Diego, USA) and ground-level ozone (O_3_) (M 400 monitor; Teledyne API, San Diego, USA). Particle number concentrations (PNC), particle area concentrations (PAC) and particle volume concentrations (PVC) were derived from measurements of the particle size distribution (size range 10–700 nm) using a Differential Mobility Particle Sizer (DMPS, custom built) [33].As UFP (by definition particles <100 nm) typically dominate ambient total PNC (here in the size range 10–700 nm), we use PNC as a proxy for UFP. The coarse fraction of PM (PM_10-2.5_) was calculated by subtracting the PM_2.5_ level from that of PM_10_. Temperature and relative humidity (hourly measurements) were measured with the HMP45a probe (Vaisala, Helsinki).

For PM_10_ and PM_2.5_ additional measurements with the beta attenuation method in 24 hour resolution were performed with SM200 instruments (OPSIS AB; Furulund, Sweden). These measurements are close to the reference method proposed by the EU and typically yield higher mass values than the TEOM method because of loss of volatile material from the latter. Daily averages (midnight to midnight) were derived from the 1-hour data. Missing values were not imputed.

### Ethics

The study adheres to the standards of the Danish Data Protection Agency. No ethical approval is required for retrospective register studies in Denmark.

### Statistical analysis

The time-stratified case-crossover design was applied to investigate the association between air pollution and OHCA. The case-crossover design was developed as a variant of the case-control design to study the effects of transient exposures on emergency events, comparing each person's exposure in a time period just prior to a case-defining event with person's exposure at other times [34]. Hereby, control on all measured and unmeasured personal characteristics that do not vary over a short time period is accomplished. If in addition, the control days are chosen close to the event day, personal characteristics that vary slowly over time are also controlled by matching. A time-stratified approach was applied to select the control days, defining the day of OHCA as the case day and same day of the week in the same month and year as control days. This approach was also applied in other studies [22]–[27]. With this approach even very strong confounding of exposure by seasonal patterns is controlled by design [35]–[38]. The data were analysed using conditional logistic regression analysis (PROC PHREG in SAS 9.2, SAS Institute, Cary, NC).

Public holidays were controlled for by use of a binary variable. Previous studies in Copenhagen reported a linear relationship between the air pollutants and the cause-specific admissions for the period 1999–2006 [39], [40]. The pollutants were therefore modelled as linear terms, one pollutant at a time.

Lag0 (same day exposure as day of admission) to lag5 (exposure five days prior to day of admission) were investigated, as well as cumulative averages: mean of lag0–1 (2-day moving average, CA2), and up to mean lag0–4 (CA5). Control days for the lags were defined as for lag0. The values of the cumulative averages were set as missing if any of the values needed for computing them were missing. All models included a single lag.

For analyses of the hourly lags, the exposure during the hour in which the OHCA occurred was designated lag0, e.g. if the OHCA occurred at 14∶35 the pollutant level at 14∶00 was used as lag0. Hourly lags of lag1 to lag7, and cumulative average exposure of CA4 (mean of lag0–3), CA8 and CA24 were also investigated. For the cumulative average exposures (CA4, CA8, CA24), values were considered missing if <75% of the hours needed for the average were available. This hourly lag structure was applied in a previous study [25]. All models included a single lag.

Although intra-individual factors cannot be examined due to the nature of the case-crossover design where each person is his/her own control, inter-individual variation using an interaction term between the susceptibility variable and a pollutant in the conditional logistic regression model yields the possibility to detect a p-value for interaction and when significant the subgroup specific estimates are valid. Susceptibility was therefore investigated in stratified analyses by age and sex, followed by models with interaction terms. Age was categorised as 19–65, 66–75 and >75 years.

Odds ratios (OR) and the 95% confidence intervals (CI) were calculated per inter-quartile range (IQR) increase in pollutant levels, which provide the magnitude-of-risk estimates that are comparable across the pollutants. The results are presented as the per cent excess risk in OHCA per IQR increase in a pollutant (on case days) using the following calculation: (exp^(βxIQR)^−1)×100%, where β is the model estimate.

For analysis of a given lagged exposure, a case was dropped automatically if exposure and meteorological data were not available for the case and at least one control day.

The difference in pollution levels, temperature and relative humidity for each of the 4 657 OHCA cases on a case day and control days was calculated. As mentioned above, a time-stratified approach was applied to select the control days, defining the day of OHCA as the case day and same day of the week in the same month and year as control days. This means that there were theoretically 3 to 4 control days per case day, hence the average pollution, temperature and relative humidity value of the control days was calculated and then subtracted from the value on the case day. The average of these differences and 95% CI were estimated for the 4 657 OHCA cases in SAS with the PROC MEANS command and CLM as option (SAS 9.2, SAS Institute, Cary, NC).

Sensitivity analyses were applied. The linearity of the relationship between OHCA and temperature and relative humidity was confirmed in generalised additive Poisson time-series regression models (GAM) with the use of the *gam* procedure, *mgcv* package in R statistical software (R Development Core Team, 2010). Smoothing splines of calendar time with 1 to 4 degrees of freedom per year (df/year) were used to control for long-term trend and seasonality. Models were run with linear and non-linear terms of lag3 of temperature and lag3 of relative humidity, the latter as a smoothing spline function with 3, 5 and 7 df. We investigated whether the non-linear terms of temperature and relative humidity improved the models by conducting log-likelihood ratio tests, i.e. compare the model with the linear term with that of the non-linear term. We decided to use linear terms for temperature and relative humidity, as the splines were insignificant, did not add value to the models and the pollutant model estimates were not influenced, whether 3, 5 or 7 df (Figures S1 in File S1). The GAM models were also adjusted for lag3 of temperature or lag3 of relative humidity, lag3 of PM_10_, day of the week and public holidays. We only ran GAM models adjusted for lag3 of PM_10_ as none of the other lags or pollutants (except PM_2.5_) were significantly associated with OHCA, and the association between PM_2.5_ and OHCA was similar to that of PM_10_.

As mentioned above, data were available for PM_2.5_ and PM_10_ that were recorded with two detection systems (TEOM and the beta attenuation system). The association between OHCA and PM_2.5_ and PM_10_ measured with these two detection systems were compared.

Toxicological studies reported that O_3_may react with the surface of particles rendering them more biologically reactive [41]. Although the association between OHCA and O_3_ was not significant at any lag, it was worthwhile to investigate whether O_3_ may alter the association between OHCA and PM_2.5_ and PM_10_.

The analyses of the different lags were performed individually. Since there is a strong correlation between the levels of a pollutant on days close to each other (i.e. different lags), other studies applied constrained distributed lag models to overcome this problem [42], [43]. We ran constrained second degree polynomial distributed lag models for the daily lags that included lag0 to lag5 of either PM_2.5_or PM_10_ [42], [43]. The models were adjusted for the distributed lag0 to lag5 of temperature, relative humidity, public holiday, day of the week and month-year strata. We used the R statistical software (R Development Core Team, 2010). Unconstrained distributed lag conditional logistic regression models were also applied, i.e. adding lag0 to lag5 together in one model and summing the model estimates to get a cumulative estimate. We did not run distributed lag models for the hourly lags or the other pollutants as none displayed significant associations with OHCA.

## Results

Table 1 indicates the characteristics of the 4 657 OHCA events during the study period. The majority of OHCA events occurred among men and those older than 75 years. OHCA events occurred more frequently after waking up in the morning, as can be seen by the steep increase between 7am–9am (Figure S2 in File S1). Figure S3 in File S1 is a time-series of the daily number of OHCA events during the study period. The number of OHCAs varied from 0 to 8 per day.

**Table 1:** Characteristics of the out-of-hospital cardiac arrests in Copenhagen (1 January 2000–31 December 2010).

	No. participants	%
**Total**	4657	100.0
**Alive 30 days after event**		
No	1756	37.7
Yes	252	5.4
Not sure	2649	56.9
**Sex**		
Male	2811	60.4
Female	1846	39.6
**Age**		
<60 years	1252	26.9
60–75 years	1410	30.3
>75 years	1995	42.8

Tables 2 and 3 provide an overview of the 1-hour and daily air pollution, temperature and relative humidity data, respectively. For particulates especially there were many missing values due to later start of monitoring by TEOM and technical problems (PNC) during the study period. PM_2.5_ levels were quite constant during the day (all days of the week) and there was no obvious increase during morning or afternoon rush hour traffic on weekdays (7am–9am and 4pm–7pm) (Figure S4 in File S1). On weekdays PM_10_ and PM_10-2.5_ levels increased between 4am–6am, then remained constant until around 2pm followed by a slow decrease during the rest of the day (Figures S5 and S6 in File S1). PNC levels (a proxy for UFP) earlier in the day coincide with rush hour (Figure S7 in File S1) and the peaks of NO_2_, NO_x_ and CO (results not shown). A peak in PNC levels was also observed at noon during June and October.

**Table 2:** Descriptive statistics for daily air pollutant and meteorological levels (lag0) on days that out-of-hospital cardiac arrests occurred in Copenhagen (1 January 2000–31 December 2010).

					Percentiles				Difference between case days and mean control days (95% CI)^b^
	No. days^a^	No. days missing data	Mean	SD	25^th^	50^th^	75^th^	IQR	
**PM_10_ (µg/m^3^)**	2753	962	15.34	8.74	10.57	13.49	17.79	7.22	−0.04 (−0.39; 0.30)
**PM_2.5_ (µg/m^3^)**	2753	1062	10.16	5.31	6.88	8.82	11.57	4.69	−0.11 (−0.34; 0.11)
**PM_10-2.5_ (µg/m^3^)**	2753	1348	4.67	6.55	2.21	3.93	5.95	3.74	0.08 (−0.21; 0.36)
**PAC (µm^2^/m^3^)**	2753	1372	202.13	129.20	110.55	174.01	265.55	155.00	−5.14 (−10.91; 0.64)
**PVC (µm^3^/m^3^)**	2753	1372	8.31	6.29	3.88	6.60	11.02	7.14	−0.27 (−0.55; 0.02)
**PNC (no./cm^3^)**	2753	1372	6405	3210	4138	5749	7967	3828	−33 (−163; 97)
**NO_x_(ppb)**	2753	128	14.00	7.89	8.59	12.05	17.09	8.50	−0.18 (−0.42; 0.06)
**NO_2_ (ppb)**	2753	128	11.05	4.86	7.45	10.31	13.87	6.42	−0.10 (−0.25; 0.05)
**O_3_ (ppb)**	2753	973	25.93	9.88	19.14	26.23	32.59	13.44	0.13 (−0.15; 0.40)
**CO (ppm)**	2753	152	0.287	0.168	0.201	0.254	0.321	0.12	−0.003 (−0.005; 0.000)
**Temperature (°C)**	2753	92	9.00	6.80	3.68	8.50	14.52	10.84	−0.04 (−0.13; 0.05)
**Relative humidity (%)**	2753	92	73.37	10.97	66.10	74.49	81.47	15.36	−0.14 (−0.42; 0.14)

SD: Standard deviation.

IQR: Interquartile range.

a Of the 4018 days during the study period, 4657 OHCAs occurred on 2753 days.

b Differences between case days and control days are calculated by subtracting the average of the level on the associated control days from the case day. The average of these differences for the 4657 OHCA cases is then calculated.

**Table 3:** Descriptive hourly air pollutant and meteorological data (lag0) on days that out-of-hospital cardiac arrests occurred in Copenhagen (1 January 2000–31 December 2010).

					Percentiles				Difference between case days and mean control days (95% CI)^b^
	No. hours^a^	No. hours missing data	Mean	SD	25^th^	50^th^	75^th^	IQR	
**PM_10_ (µg/m^3^)**	4556	1669	15.73	12.05	9.65	13.35	18.60	8.95	−0.03 (−0.51; 0.45)
**PM_2.5_ (µg/m^3^)**	4556	1878	10.31	8.52	6.20	8.73	12.10	5.90	−0.01 (−0.37; 0.35)
**PM_10-2.5_ (µg/m^3^)**	4556	2347	4.84	9.38	1.45	3.75	6.55	5.10	0.05 (−0.38; 0.47)
**PAC (µm^2^/m^3^)**	4556	2393	200.74	143.26	96.18	163.92	270.89	174.71	−5.96 (−12.85; 0.93)
**PVC (µm^3^/m^3^)**	4556	2393	8.16	6.82	3.28	6.08	11.05	7.77	−0.36 (−0.69; −0.03)
**PNC (no./cm^3^)**	4556	2393	6629	4376	3619	5557	8474	4856	46 (−146; 238)
**NO_x_(ppb)**	4556	258	14.75	11.82	7.07	11.58	18.35	11.28	−0.16 (−0.52; 0.20)
**NO_2_ (ppb)**	4556	258	11.44	7.00	6.14	9.87	15.06	8.92	−0.01 (−0.22; 0.20)
**O_3_ (ppb)**	4556	1712	25.94	12.31	17.57	26.20	34.11	16.54	0.22 (−0.14; 0.58)
**CO (ppm)**	4556	295	0.296	0.187	0.195	0.250	0.333	0.138	−0.003 (−0.006; 0.001)
**Temperature (°C)**	4556	192	8.84	7.16	3.23	8.13	14.42	11.19	−0.04 (−0.14; 0.07)
**Relative humidity (%)**	4556	192	72.29	14.55	62.86	75.05	83.33	20.47	−0.19 (−0.56; 0.18)

SD: Standard deviation.

IQR: Interquartile range.

a Of the 96432 hours (on 4018 days) during the study period, 4657 OHCAs occurred on 4556 hours (on 2753 days).

b Differences between case hours and control hours are calculated by subtracting the average of the level on the associated control hours from the case hour. The average of these differences for the 4657 OHCA cases is then calculated.

The daily WHO and EU air quality limits for PM_10_ (50 µg.m^−3^) were exceeded on 12 days at the urban background level (Figure S8 in File S1) [32]. The daily WHO air quality limit for PM_2.5_ (25 µg.m^−3^) was exceeded on 44 days (Figure S9 in File S1) [32]. The WHO and EU air quality limits for NO_2_ 105 ppb (1-hour max) were not exceeded [32]. The 1-hour and daily level of a pollutant on the case day was not significantly different from those on the control days (Tables 2 and 3, Table S1 in File S2).

Tables 4 and 5 display the Spearman correlations between the 1-hour and daily averages of the air pollutants, temperature and relative humidity, respectively. The strongest correlations were between PM_10_, PM_2.5_, PAC and PVC. PM_10-2.5_ had a stronger correlation with PM_10_ than PM_2.5_. O_3_ had an inverse correlation with the other pollutants (the strongest with NO_x_), except with PM_10-2.5_. This is partly due to consumption of O_3_ by diesel engine emissions of NO in the urban areas and partly due to seasonal patterns with maximum levels of ozone in the summer, whereas the other pollutants peak during winter.

**Table 4:** Spearman correlation coefficients between exposure variables (daily lag0) on days that out-of-hospital cardiac arrests occurred in Copenhagen (1 January 2000–31 December 2010).

	PM_2.5_	PM_10-2.5_	PAC	PVC	PNC	NO_x_	NO_2_	O_3_	CO	Temp	Rel. hum
**PM_10_**	0.812	0.589	0.591	0.592	0.373	0.303	0.323	0.028^a^	0.216	0.245	−0.050
	1405	1405	1039	1039	1039	1738	1738	1462	1697	1776	1776
**PM_2.5_**		0.100	0.759	0.788	0.338	0.368	0.397	−0.107	0.371	0.038^a^	0.160
		1405	1090	1090	1090	1637	1637	1650	1633	1665	1665
**PM_10-2.5_**			−0.036^a^	−0.084	0.174	0.061	0.058	0.290	−0.197	0.499	−0.352
			871	871	871	1355	1355	1370	1351	1390	1390
**PAC**				0.973	0.678	0.490	0.528	−0.200	0.477	−0.036^a^	0.225
				1381	1381	1348	1348	1111	1355	1362	1362
**PVC**					0.538	0.463	0.501	−0.261	0.491	−0.093	0.330
					1381	1348	1348	1111	1355	1362	1362
**PNC**						0.445	0.468	−0.015^a^	0.325	0.071	−0.052
						1348	1348	1111	1355	1362	1362
**NO_x_**							0.981	−0.548	0.587	−0.125	0.278
							2625	1733	2542	2584	2584
**NO_2_**								−0.520	0.599	−0.134	0.274
								1733	2542	2584	2584
**O_3_**									−0.476	0.362	−0.654
									1695	1754	1754
**CO**										−0.488	0.394
										2560	2560
**Temp**											−0.351
											2661

a p-value>0.05, otherwise p-value<0.05.

Top value is the Spearman correlation coefficient and the bottom value is the number of days. Of the 4018 days during the study period, 4657 OHCAs occurred on 2753 days.

**Table 5:** Spearman correlation coefficients between exposure variables (hourly lag0) on days that out-of-hospital cardiac arrests occurred in Copenhagen (1 January 2002–31 December 2010).

	PM_2.5_	PM_10-2.5_	PAC	PVC	PNC	NO_x_	NO_2_	O_3_	CO	Temp	Rel. hum
**PM_10_**	0.777	0.591	0.584	0.580	0.409	0.328	0.343	−0.066	0.264	0.192	−0.080
	2209	2209	1619	1619	1619	2779	2779	2314	2712	2854	2854
**PM_2.5_**		0.038	0.730	0.750	0.397	0.370	0.403	−0.218	0.382	0.020^a^	0.147
		2209	1695	1695	1695	2575	2575	2599	2565	2626	2626
**PM_10-2.5_**			−0.027^a^	−0.071	0.165	0.090	0.072	0.207	−0.126	0.412	−0.334
			1330	1330	1330	2116	2116	2147	2105	2182	2182
**PAC**				0.975	0.689	0.544	0.579	−0.331	0.529	−0.059	0.238
				2163	2163	2094	2094	1728	2115	2134	2134
**PVC**					0.557	0.496	0.529	−0.366	0.534	−0.111	0.323
					2163	2094	2094	1728	2115	2134	2134
**PNC**						0.585	0.609	−0.186	0.407	0.055	−0.011^a^
						2094	2094	1728	2115	2134	2134
**NO_x_**							0.975	−0.622	0.615	−0.134	0.246
							4298	2757	4157	4215	4215
**NO_2_**								−0.620	0.623	−0.139	0.260
								2757	4157	4215	4215
**O_3_**									−0.492	0.365	−0.626
									2695	2792	2792
**CO**										−0.399	0.308
										4179	4179
**Temp**											−0.351
											4364

a p-value>0.05. otherwise p-value<0.05.

Top value is the Spearman correlation coefficient and the bottom value is the number of hours. Of the 96432 hours (on 4018 days) during the study period, 4657 OHCAs occurred on 4556 hours (on 2753 days).

Figures S10 to S13 in File S1 illustrate the % change in the OHCA events per IQR increase in the daily and hourly lags of the pollutants, respectively, after adjusting for public holidays, temperature and relative humidity in single pollutant models. The same lag of the pollutants, temperature and relative humidity was included in each model. None of the hourly lags of any of the pollutants were significantly associated with OHCA (Figures S10 and S11 in File S1). The strongest association was observed between the daily lag3 of PM_10_, lag4 of PM_2.5_, lag3 of PM_10-2.5_, lag3 of NO_x_ and lag4 of CO (Figures S12 and S13 in File S1). An IQR increase in lag3 of PM_10_ and PM_2.5_ was associated with a significant increase of 5% (95% CI: 1%; 9%) and 4% (95% CI: 0%; 9%) in OHCA events, respectively (Table 6). None of the other daily lags or other pollutants was significantly associated with OHCA. PM_10-2.5_ (lag3) had a slightly weaker association with OHCA than PM_10_ and PM_2.5_, although not significant.

**Table 6:** Association between air pollutants (single pollutant models) and out-of-hospital cardiac arrests in Copenhagen, expressed as percentage increase in risk (%) and 95% confidence intervals per inter-quartile increase in daily lag0 to lag5 and 2-day, 4-day and 6-day cumulative average (1 January 2000–31 December 2010).

	PM_2.5_					PM_10_					PM_10-2.5_					NO_x_				
	IQR	n^a^	%	95% CI		IQR	n^a^	%	95% CI		IQR	n^a^	%	95% CI		IQR	n^a^	%	95% CI	
**Lag0**	5	2758	−1.4	−5.5	2.9	7	2996	0.3	−3.2	3.9	4	2326	0.8	−2.1	3.8	9	4388	−3.2	−7.3	1.1
**Lag1**	5	2774	−0.7	−4.7	3.6	7	3002	2.4	−1.2	6.2	4	2345	2.1	−1.1	5.3	9	4395	1.2	−3.0	5.6
**Lag2**	5	2783	2.4	−1.8	6.8	7	3005	2.1	−1.3	5.7	4	2352	2.4	−0.4	5.4	9	4386	−1.9	−6.0	2.5
**Lag3**	5	2777	**4.4**	**0.2**	**8.8**	7	3000	**4.7**	**0.7**	**8.8**	4	2348	3.5	−0.3	7.4	9	4391	3.4	−0.9	7.9
**Lag4**	5	2777	**5.2**	**1.0**	**9.5**	7	3002	**3.8**	**0.2**	**7.6**	4	2350	1.9	−1.3	5.1	9	4388	1.9	−2.4	6.3
**Lag5**	5	2775	−0.5	−4.5	3.7	7	3006	1.8	−1.9	5.6	4	2355	2.1	−1.2	5.5	9	4386	1.2	−3.1	5.7
**CA2**	4	2728	−1.3	−5.0	2.6	6	2961	1.2	−2.4	4.8	3	2291	1.4	−1.3	4.2	8	4337	−1.1	−5.6	3.7
**CA4**	4	2651	1.3	−3.3	6.2	6	2890	3.8	−0.7	8.5	3	2212	3.3	−0.3	7.1	7	4247	−0.3	−5.3	5.0
**CA6**	4	2576	2.7	−2.7	8.4	5	2817	3.9	−0.5	8.4	3	2133	3.3	−0.9	7.6	6	4153	1.1	−4.0	6.6

a Number of OHCA cases used in the models, which is less than 4657 due to missing exposure data.

Lag3 of PM_10_, PM_2.5_, PM_10-2.5_ and NO_x_ was selected to investigate susceptibility (Table 7). Although a stronger association was observed in the stratified analyses between OHCA, and PM_10_, PM_2.5_ and PM_10-2.5_ for men and between OHCA and NO_x_ for women, the interaction terms were not significant (p>0.05).

**Table 7:** Association between air pollutants (single pollutant models) and out-of-hospital cardiac arrests in Copenhagen by subgroups, expressed as percentage increase in risk (%) and 95% confidence intervals per inter-quartile increase in daily lag3 (1 January 2000–31 December 2010).

	PM_2.5_					PM_10_					PM_10-2.5_					NO_x_				
	IQR	n^a^	%	95% CI		IQR	n	%	95% CI		IQR	n^a^	%	95% CI		IQR	n	%	95% CI	
**All**	5	2777	**4.4**	**0.2**	**8.8**	7	3000	**4.7**	**0.7**	**8.8**	4	2348	3.5	−0.3	7.4	9	4391	3.4	−0.9	7.9
**Sex**																				
Male	5	1688	**6.2**	**0.8**	**11.9**	7	1816	**7.9**	**2.6**	**13.4**	4	1428	**7.4**	**2.0**	**13.1**	9	2647	0.5	−4.8	6.1
Female	5	1089	1.8	−5.1	9.1	7	1184	−0.6	−7.0	6.1	4	920	−2.6	−9.5	4.8	9	1744	**8.9**	**1.7**	**16.7**
**Age**																				
<60 years	5	783	6.6	−1.4	15.2	7	838	6.1	−1.0	13.7	4	671	6.4	−0.6	13.8	9	1173	5.6	−2.8	14.7
60–75 years	5	857	1.6	−6.0	9.9	7	910	4.1	−3.3	12.2	4	722	3.4	−3.7	11.0	9	1333	−1.3	−8.6	6.7
>75 years	5	1137	5.2	−1.3	12.2	7	1252	3.5	−2.7	10.2	4	955	−0.7	−8.1	7.2	9	1885	5.9	−0.8	13.1

a Number of OHCA cases used in the models, which is less than 4657 due to missing exposure data.

In Copenhagen, monitoring of PM_10_ and PM_2.5_ by beta attenuation started in May 2002 and April 2008, respectively (Figures S14 and S15 in File S1, Table S2 in File S2), whereas PM_10_ and PM_2.5_ measurements by TEOM started in 2002 and 2003, respectively (Figures S8 and S9 in File S1, Table S2 in File S2). In a sensitivity analyses, we observed similar associations between OHCA and lag3 of PM_10_ measured with the TEOM and beta attenuation system for the study period 1 May 2002–31 December 2010 (Figure S16 in File S1, Table S3 in File S2). We did not observe a similar association between OHCA and lag3 of PM_2.5_ measured with the two detection systems for the study period 1 April 2008–31 December 2010 (Figure S17 in File S1, Table S4 in File S2). The correlation between PM_10_ measured with the two detection systems was stronger than that of PM_2.5_: 0.843 for PM_10_ over 1 770 days and 0.771 for PM_2.5_ over 454 days (Tables S5 and S6 in File S2).

In a sensitivity analyses, we observed that further adjustment for O_3_ had minimum influence, i.e. slightly increased the strength of associations between OHCA and the PM measures, except for PM_2.5_ at lag3 (Table 6, Figure S18 in File S1, Table S7 in File S2).

The cumulative odds ratio for lag0 to lag5 of PM_10_ (obtained in the constrained second degree polynomial distributed lag model) (1.050 (95% CI: 0.990–1.118)) was similar to that of lag5 (1.018 (95% CI: 0.981–1.056)) and CA6 (1.039 (95% CI: 0.995–1.084)) of PM_10_ (obtained in the single lag conditional logistic regression models) (Table 6, Figure S19 in File S1). The cumulative odds ratio for lag0 to lag5 of PM_10_ (obtained in the unconstrained conditional logistic regression model) was 1.031. The cumulative odds ratio for lag0 to lag5 of PM_2.5_ (1.000 (95% CI: 0.943–1.060)) was also similar to lag5 (0.995 (95% CI: 0.955–1.037)) and CA6 (1.027 (95% CI: 0.973–1.084)) (Table 6, Figure S19 in File S1). The cumulative odds ratio for lag0 to lag5 of PM_2.5_ (obtained in the unconstrained conditional logistic regression model) was 1.010.

## Discussion

In this case-crossover study from Copenhagen, Denmark, we found a 4% and 5% increase in OHCA events for an IQR increase in lag3 of PM_2.5_ and PM_10_, respectively. We found no evidence for effects occurring within hours or any significant associations with other pollutants such as the PNC (a proxy for UFP), PAC, PVC, NO_2_, NO_x_ and CO or O_3_.

In Copenhagen PM_2.5_ is dominated by long range transport and the levels are rather uniform across the city with limited diurnal variation. PM_10_ and PM_10-2.5_ tracked traffic rush hour quite well in Copenhagen. This traffic-related coarse fraction could be rich in transition metals due to wear and tear of car and truck breaks. Recent in vivo data suggest that transition metals can catalyse an oxidative stress reaction in the lung, leading to inflammatory lung injury [8], [9] and increased arrhythmia [9], [10]. Moreover, compositional analyses of ambient air in Quebec suggest that particulate matter with high sulphate fractions is more strongly associated with increased hospitalisations for cardiac and respiratory diseases [44].

From a mechanistic point of view UFP would be expected to be the strongest risk factors for OHCA. UFP have a high surface area and contain carbon, polycyclic aromatic hydrocarbons and metals [45]. UFP are capable of penetrating the pulmonary interstitium, causing interstitial inflammation and significant oxidative stress [46], [47], may pass into the blood circulation [48] and possibly induce endothelial dysfunction as well as have prothrombotic and arythmogenic effects [49], [50]. Indeed, a previous study from Copenhagen found associations between high UFP levels and admissions for ischemic stroke [51]. Ischemic stroke is mainly a thrombotic event to some extent and similar to myocardial infarction, which is the dominant cause of cardiac arrest. Similarly, a study from Rome with model based assessment of UFP levels found associations with OHCA [24].

The lack of association between OHCA and UFP in our study could be due to high spatial variability even within our 5 km radius from the monitor. This may lead to substantial exposure misclassification. Moreover, UFP measured at the monitoring station could have other sources than traffic, as suggested by peak levels at midday during June and October. Peak levels earlier in the day coincide with rush hour and the peaks of gaseous traffic-related pollutants.

The observed effects occurring within three days is compatible with other studies in Copenhagen, i.e. emergency CVD and acute myocardial infarction hospital admissions [40], . The six international studies that investigated OHCA and daily air pollution exposure reported lag structures of lag0 to lag3 and CA2, with significant lags varying from lag0 to lag2 and CA2. Nevertheless, it is plausible that increased levels of PM_10_ or PM_2.5_ three days prior to an event results in oxidative stress and the induction of inflammation in the lungs of vulnerable persons [11]–[16], which in turn is followed by amplification of systemic pro-inflammatory cytokine levels and endothelial vasoconstrictors. A slightly slower development of such PM induced effects in Copenhagen compared to the other studies cities might be due to differences in composition of the PM or in exposure condition e.g. related to housing or outdoor activities. These biological changes may lead to changes in heart rate and blood pressure or amplification of the release of local inflammatory mediators and increased recruitment of T lymphocytes and monocytes, which in turn may result in plaque rupture and arrhythmia [8]–[10], [53], [54]. A study from Copenhagen reported that reduction of the home indoor levels of PM_2.5_ and UFP by filtration improved microvascular function within 48 hours among elderly [55]. Such an effect has been confirmed in a Canadian study with air filtration in the homes of younger healthy subjects [56].

Three of the six international studies reported significant associations between OHCA events and PM_10_ and PM_2.5_ levels. Our effect estimates of PM_10_ and PM_2.5_ are similar to those from studies conducted in Rome and Melbourne [24], [26]. The study from Rome reported a significant effect of 6% per IQR increase in PM_10_ (30 µg.m^−3^, CA2). The study from Melbourne is the first to suggest an effect of PM_2.5_: 4% per IQR increase (4 µg.m^−3^, CA2). The study also observed a 3% increase risk in OHCA per IQR increase in PM_10_ (10 µg.m^−3^, CA2) [26]. The study from New York, USA reported a significant effect of 6% per 10 µg.m^−3^ increase in the CA2 of PM_2.5_ [27]. Unlike ours, the study from Rome reported an 8% increase in OHCA deaths per IQR increase in PNC (27 790 particles.m^−3^, CA2) [24].

O_3_ may react with the surface of particles rendering them more biologically reactive [41]. We observed that adjustment for O_3_ slightly increased the association between OHCA and PM_2.5_ and PM_10_. We did not investigate possible effect modification (i.e. possible synergism) between O_3_ and the PM measures, because the O_3_ levels are rather low in Copenhagen and the number of cases included in the models is also fewer than in the single pollutant models due to missing data for the PM measures and O_3_. There is increasing interest in managing environmental air quality using multi-pollutant strategies targeted at lowering the aggregate health burden of air pollution [57]. Therefore, from both the public health and regulatory perspective, the potential for synergy among mixture of components is a particular concern. A review concluded that synergisms involving O_3_ have been demonstrated by laboratory studies of humans and animals [58]. These laboratory studies focused on subclinical outcomes (e.g. biomarkers of inflammation, oxidative stress, and so forth) observed at exposure levels much higher than typically encountered in the ambient environment and it is unclear whether synergism would manifest at clinical and public health scales. A convincing explanation of how inhaled particulate or gaseous pollutants might induce systemic vascular and cardiac molecular alterations still remains an area of on-going research [59]. Reviews highlighted that epidemiological study designs have limited ability to address the issue of synergism explicitly [58], [60], [61]. The American Heart Association also concluded that more research is needed to make robust conclusions regarding the independent cardiovascular risks posed by gaseous pollutants [7].

Two studies in Washington State, USA did not find any association between OHCA and PM_2.5_, PM_10_, SO_2_ or CO [22], [23]. The study from Rome found no association with out-of hospital cardiac *deaths* and NO_2_ or O_3_ [24]. A study from Indianapolis, USA did not find any association with PM_2.5_ on the day of the OHCA or 1–3 days before the OHCA [25]. The study from Melbourne found no association with NO_2_, SO_2_ nor O_3_ [26].

Few studies explored the association between hourly exposure and cardiac events. Contrary to our study, a study from Indianapolis, USA observed an effect of 16% increase in OHCA events per 10 µg.m^−3^ increase in PM_2.5_ within the hour of an OHCA [25]. A study from Boston, USA reported that myocardial infarction was associated with exposure in traffic 1–3 hours before the event [62]. A study from Germany reported a significant association between exposure to traffic and the onset of a myocardial infarction within one hour afterward [63]. A study from Boston, USA reported an increased risk to elevated O_3_ levels *one hour* before a cardiac arrhythmic event [64]. The lack of association with hourly air pollution exposures in our study could be due to diurnal variation in OHCA events, with more OHCA events after waking up in the morning. In contrast, PM_10_ and PM_2.5_ levels are quite constant between 7am–9am (i.e. lag0 exposure). PM_10_ levels are increasing between 4am–6am (i.e. lag of 3 hrs before the increase in OHCA events), when people are still mostly indoors at that time. None of the other studies reported the diurnal variation of air pollution and OHCA, myocardial infarction or cardiac arrhythmic events [25], [62]–[64].

No susceptible age groups were identified in our study. The study from Melbourne reported that men, the 35–64 and the 65–74 year groups were more susceptible toPM_2.5_ [26].

Our mean age at OHCA was 69 years and is comparable to that of the Melbourne study (72 years) [26]. The study from Rome included more elderly persons (67% older than 74 years) [24]. This may be one of the reasons for the observed heterogeneity in results. Copenhagen had similar air pollution levels than Melbourne, but 2–3 times lower than Rome [24]. Other reasons may be related to different PM composition, different sources, climate, behaviour and building traditions, which may influence indoor infiltration and exposure of air pollution.

We performed the analyses of the different lags individually in separate conditional logistic regression models. Since there is a strong correlation between the levels of a pollutant on days close to each other (i.e. different lags), other studies applied constrained distributed lag models to overcome this problem [42],[43]. However, we observed similar results in the single lag conditional logistic regression models, constrained second degree polynomial distributed lag models and unconstrained conditional logistic regression models for PM_10_ and PM_2.5_.

Advantages of our study include accurate meteorological, air pollution and OHCA data. The diurnal variation of OHCAs in Copenhagen is natural and has been reported in a review [65]. Moreover, case ascertainment is optimum because a physician-staffed MECU is always deployed whenever an OHCA is suspected – along with the standard ambulance. Only in the event of more than one or two OHCAs occurring simultaneously during night or day time, respectively, there could be a chance of missing OHCA cases, as an ambulance could arrive at an OHCA patient without activating the MECU. However, with approximately 300–350 OHCAs occurring in central Copenhagen yearly, the probability for this to happen is very small. Thus the number of OHCA cases that are being missed is very small. All OHCA cases were controlled every year concerning right classification (that is classified as OHCA and not for example unconscious of other causes). Misclassification, however, was not a problem since the classification occurred every day when the treating physician entered the OHCA variables in the database. The time of OHCA was defined as the time the EDC received an OHCA call from a bystander. There was no information on the estimated time period from collapse to call to the EDC, which is a time very hard to obtain and with great error in estimation

Our sample size of 4 657 OHCA events is also similar to or larger than that of the studies in Rome (n = 5 144) and Indianapolis (n = 1 374) [24]–[25]. Our study period of 11 years is longer than the studies from Rome, Indianapolis and Melbourne, which had study periods of 3–5 years [24]–[26]. We acknowledge that between only 2 576 and 3 006 cases out of the total 4 657 cases were included in the PM_10_ and PM_2.5_ models (covering around 8 to 9 years out of 11 years). This is still more than those included in the models of the Indianapolis study (1 288–1 343 cases) [24]. The studies from Melbourne and Rome did not address missing air pollution data nor reported the number of cases included in the models [25]–[26]. The missing pollutant data are mainly from the early part of the study period (January 2000 to December 2003) where exposure could be higher and different. However, few days had missing data later in the study period, which is more relevant to possible future abatement strategies.

One of the limitations of the study is the assumption that the ambient air pollution levels, temperature and humidity measured in the inner-city of Copenhagen are the same across the study area with a radius of about 5 km. The exposure error resulting from using ambient temperature and air pollution as a surrogate for personal exposure can potentially lead to bias in the estimated association, and this can be more pronounced among the elderly and other frail groups who generally spend most of their time indoors.

A second limitation is that although we excluded patients with obvious signs of death (i.e. trauma, rigor mortis, livores), we also excluded those where resuscitation was judged effortless by the emergency physician. The latter group of excluded patients could have had more severe OHCAs (i.e. more with non-shockable heart rhythm) and if the strength of the association between ambient air pollution exposure and OHCAs is increased by the severity, then our associations may be underestimated. If the patients were not resuscitated due to the late arrival of the emergency medical services (so independent of severity), then non-differential exposure misclassification occurs and our associations may be underestimated.

A third limitation is O_3_ data were collected at a monitor located in an urban setting. We are well aware that O_3_ levels in urban areas are influenced by NO emissions from traffic and that the O_3_ levels are lower than regional levels. Moreover, seasonal changes augment this difference with high levels of O_3_ in the summer, whereas NO_x_ levels are highest in the winter. This likely explains the inverse correlation between O_3_ and NO_x_/NO_2_. However, in our study the urban background O_3_ levels should reflect the exposure for the population at risk within the urbanised inner-city of Copenhagen (cases within 5 km of the urban background station) better than the regional levels measured at rural stations far from Copenhagen.

A fourth limitation is that PM_2.5_ and PM_10_ data applied in our study were recorded with the TEOM measurement system, which records lower mass than the beta attenuation system. The TEOM system records levels every 30 minutes, whereas the beta attenuation system records levels every 24 hrs. The validity of the TEOM measurement system can be affected by atmospheric conditions, particularly temperature, humidity and precipitation. In extreme cases, these effects resulted in the monitor indicating negative values. Only 11 and six of the PM_2.5_ and PM_10_values were negative in our study, respectively and were set as missing. The studies from Indianapolis and Melbourne also applied PM_2.5_ and PM_10_ data measured with TEOM [25], [26]. In a sensitivity analyses, we observed similar associations between OHCA and the daily lag3 of PM_10_ measured with the TEOM and beta attenuation system. The correlation between PM_10_ measured with the two detection systems was stronger than that of PM_2.5_. This may also explain why we did not observe a similar association between OHCA and the daily lag3 of PM_2.5_ measured with the two detection systems. Another reason could be the smaller number of OHCAs included in the PM_2.5_ models than PM_10_ models.

A fifth limitation is that information on other effect modifiers, e.g. the use of cardiac medications (beta blockers, sympathomimetics, statins), aspirin and antioxidants intake, having a pre-existing CVD, comorbidities (e.g. hypertension, COPD) along with recent lipoprotein level [18], [66]–[68], was not readily available in our study. Such effect modifiers may bias the association between the air pollutants and OHCA in either direction.

A sixth limitation is that we performed a great number of analyses for both hourly and daily lags as well as cumulative averages. We also investigated many pollutants. This amounts to a large number of tests which increases the probability of obtaining spurious significant associations (i.e. daily lag3 of PM_10_ and PM_2.5_).

In conclusion, our results support the notion that moderate increases in urban background PM_10_ and PM_2.5_ levels are associated with an increase in OHCA events. More studies are needed to clarify the effect of short-term hourly exposures.

## Supporting Information

File S1 **Supplementary figures.**
(DOC)

File S2 **Supplementary tables.**
(DOC)
