# Supplementary material for: Out-of-Hospital Cardiac Arrests and Outdoor Air Pollution Exposure in Copenhagen, Denmark
Source: PLoS One. 2013 Jan 14;8(1):e53684. doi: 10.1371/journal.pone.0053684 (PMC3544842; doi:10.1371/journal.pone.0053684)
Supplement: File S1 — Supplementary figures. (DOC) [file pone.0053684.s001.doc]

Figure S1. Smoothed relationship (expressed as the model estimate) between out-of-hospital cardiac arrests (OHCA) and a unit increase in lag3 of (a) temperature and (b) relative humidity in Copenhagen, Denmark during 2000 − 2010.

Models adjusted for lag3 of relative humidity (or lag3 of temperature), lag3 of PM_10_, day of the week and public holidays and long-term trend.

Percent excess risk in OHCA = (exp^β^ – 1) × 100%, where β is the model estimate.

Figure S2. Diurnal variation in occurrence of 4657 out-of-hospital cardiac arrests in Copenhagen during 1 January 2000 - 31 December 2010.

Figure S3. Time series of 4657 out-of-hospital cardiac arrests in Copenhagen during 1 January 2000 - 31 December 2010.

Figure S4. PM_2.5_ levels (µg.m^-3^) measured with the tapered element oscillating microbalance measurement system by hour and day of week during 1 January 2000 - 31 December 2010.

Figure S5. PM_10_ levels (µg.m^-3^) measured with the tapered element oscillating microbalance measurement system by hour and day of week during 1 January 2000 - 31 December 2010.


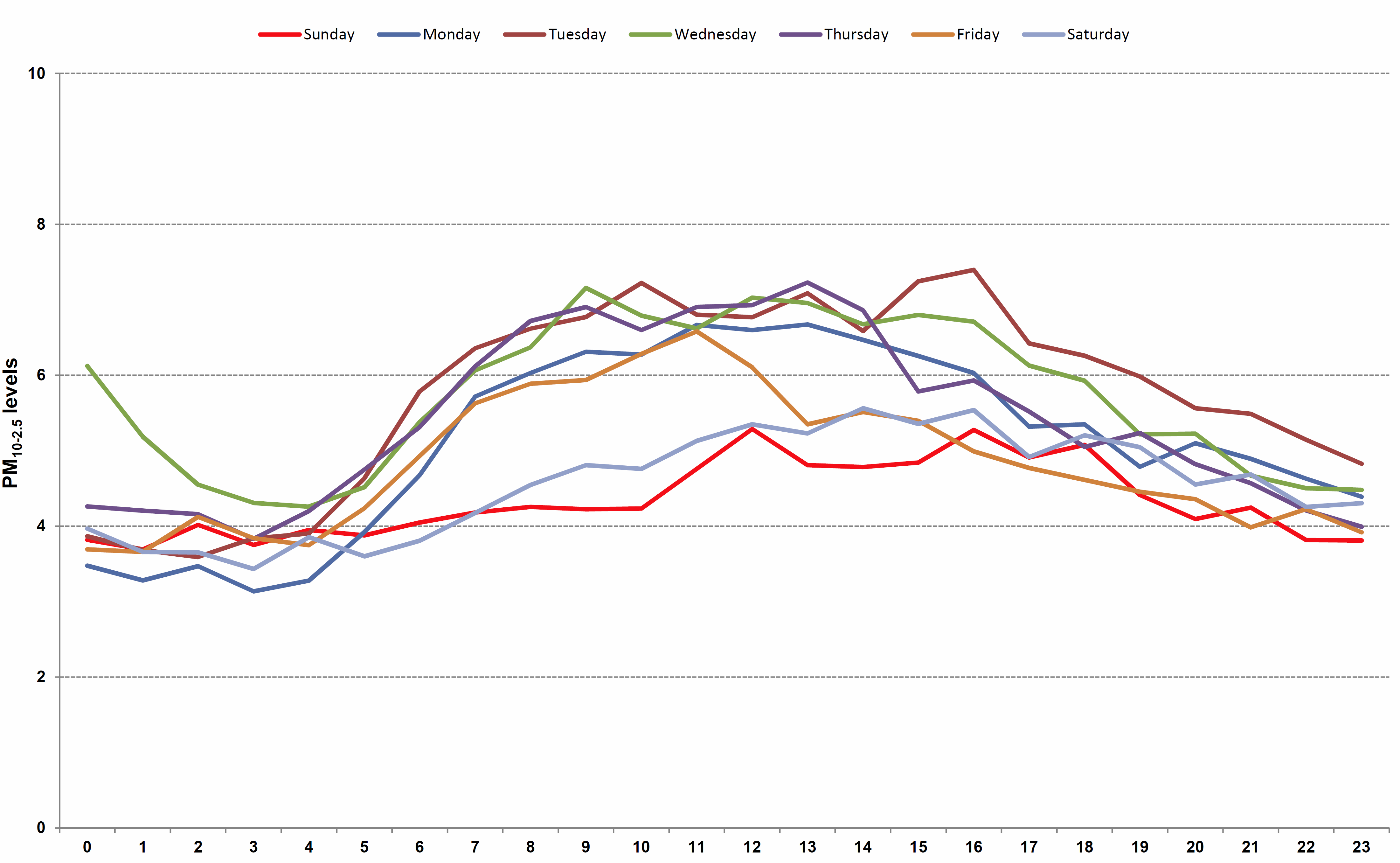


Figure S6. PM_10-2.5_ levels (µg.m^-3^) measured with the tapered element oscillating microbalance measurement system by hour and day of week during 1 January 2000 - 31 December 2010.


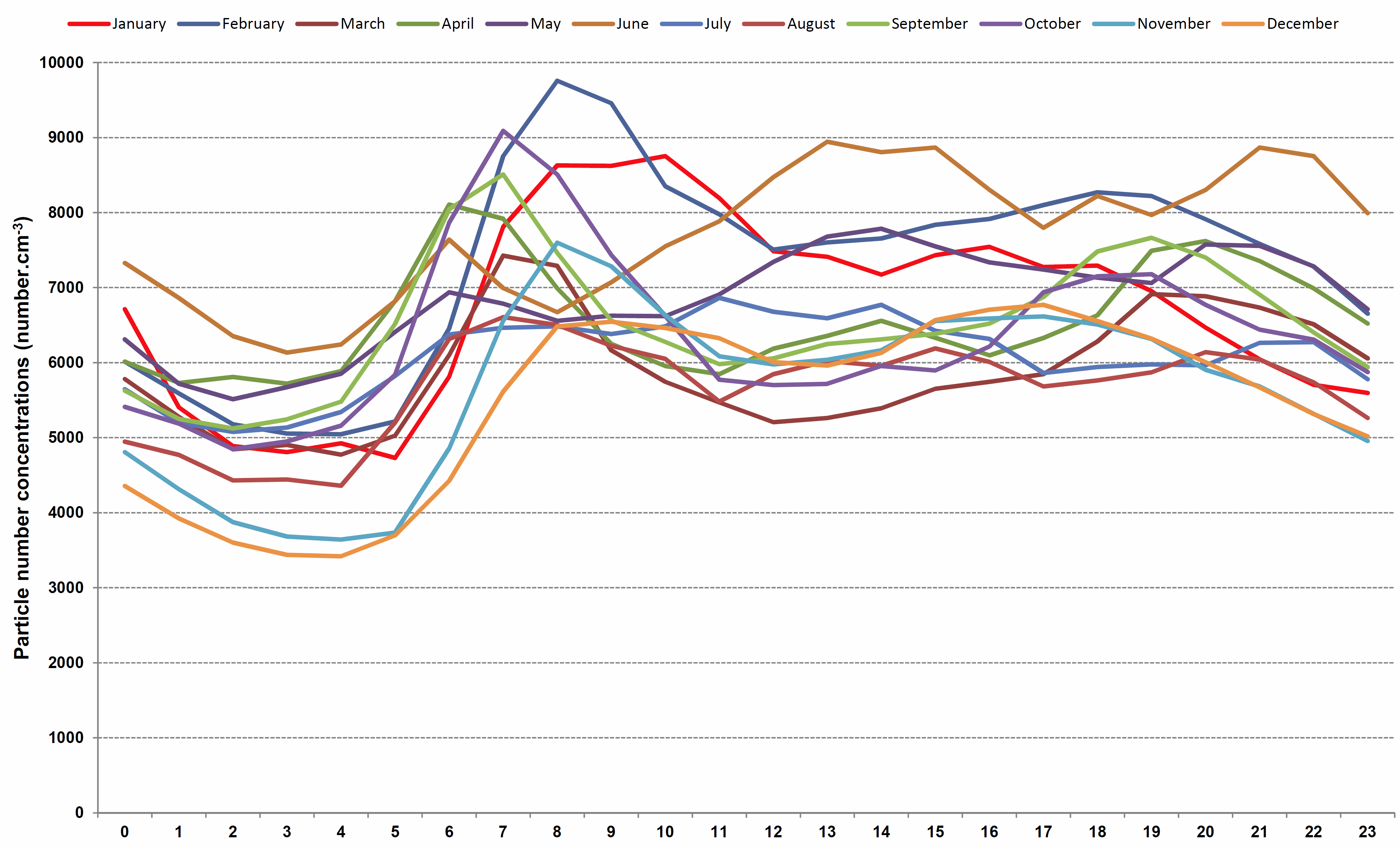


Figure S7. Particle number concentrations (number.cm^-3^) by hour and month during 1 January 2000 - 31 December 2010.

Figure S8. Time series of PM_10_ levels (µg.m^-3^) measured with the tapered element oscillating microbalance measurement system in Copenhagen during 1 January 2000 - 31 December 2010.

Figure S9. Time series of PM_2.5_ levels (µg.m^-3^) measured with the tapered element oscillating microbalance measurement system in Copenhagen during 1 January 2000 - 31 December 2010.

Figure S10. Percentage change (95% CI) in out-of-hospital cardiac arrests in Copenhagen per inter-quartile range increase in *hourly* air pollutant levels during 1 January 2000−31 December 2010, adjusted for public holidays, temperature and relative humidity (same lag as pollutant).

**n is the number of OHCA cases used in the models and is less than 4657 due to missing exposure data

Figure S11. Percentage change (95% CI) in out-of-hospital cardiac arrests in Copenhagen per inter-quartile range increase in *hourly* PM_10_, PM_2.5_ and PM_10-2.5_ levels during 1 January 2000−31 December 2010, adjusted for public holidays, temperature and relative humidity (same lag as pollutant).

**n is the number of OHCA cases used in the models and is less than 4657 due to missing exposure data

Figure S12. Percentage change (95% CI) in out-of-hospital cardiac arrests in Copenhagen per inter-quartile range increase in *daily* air pollutant levels during 1 January 2000−31 December 2010, adjusted for public holidays, temperature and relative humidity (same lag as pollutant).

**n is the number of OHCA cases used in the models and is less than 4657 due to missing exposure data

Figure S13. Percentage change (95% CI) in out-of-hospital cardiac arrests in Copenhagen per inter-quartile range increase in *daily* PM_10_, PM_2.5_ and PM_10-2.5_ levels during 1 January 2000−31 December 2010, adjusted for public holidays, temperature and relative humidity (same lag as pollutant).

**n is the number of OHCA cases used in the models and is less than 4657 due to missing exposure data

Figure S14. Time series of PM_10_ levels (µg.m^-3^) measured with the beta attenuation measurement system in Copenhagen during 1 January 2000 - 31 December 2010.

Figure S15. Time series of PM_2.5_ levels (µg.m^-3^) measured with the beta attenuation measurement system in Copenhagen during 1 January 2000 - 31 December 2010.

(a) (b)

Figure S16. Percentage change (95% CI) in 3606 out-of-hospital cardiac arrests in Copenhagen per inter-quartile range increase in *daily* PM_10_ levels measured with the (a) tapered element oscillating microbalance measurement system and (b) beta attenuation system during 1 May 2002−31 December 2010 (not 1 Jan 2000 – 31 Dec 2010), adjusted for public holidays, temperature and relative humidity (same lag as pollutant).

**n is the number of OHCA cases used in the models and is less than 3606 due to missing exposure data

(a) (b)

Figure S17. Percentage change (95% CI) in 1117 out-of-hospital cardiac arrests in Copenhagen per inter-quartile range increase in *daily* PM_2.5_ levels measured with the (A) tapered element oscillating microbalance measurement system and (B) beta attenuation system during 1 April 2008−31 December 2010 (not 1 Jan 2000 – 31 Dec 2010), adjusted for public holidays, temperature and relative humidity (same lag as pollutant).

**n is the number of OHCA cases used in the models and is less than 1117 due to missing exposure data


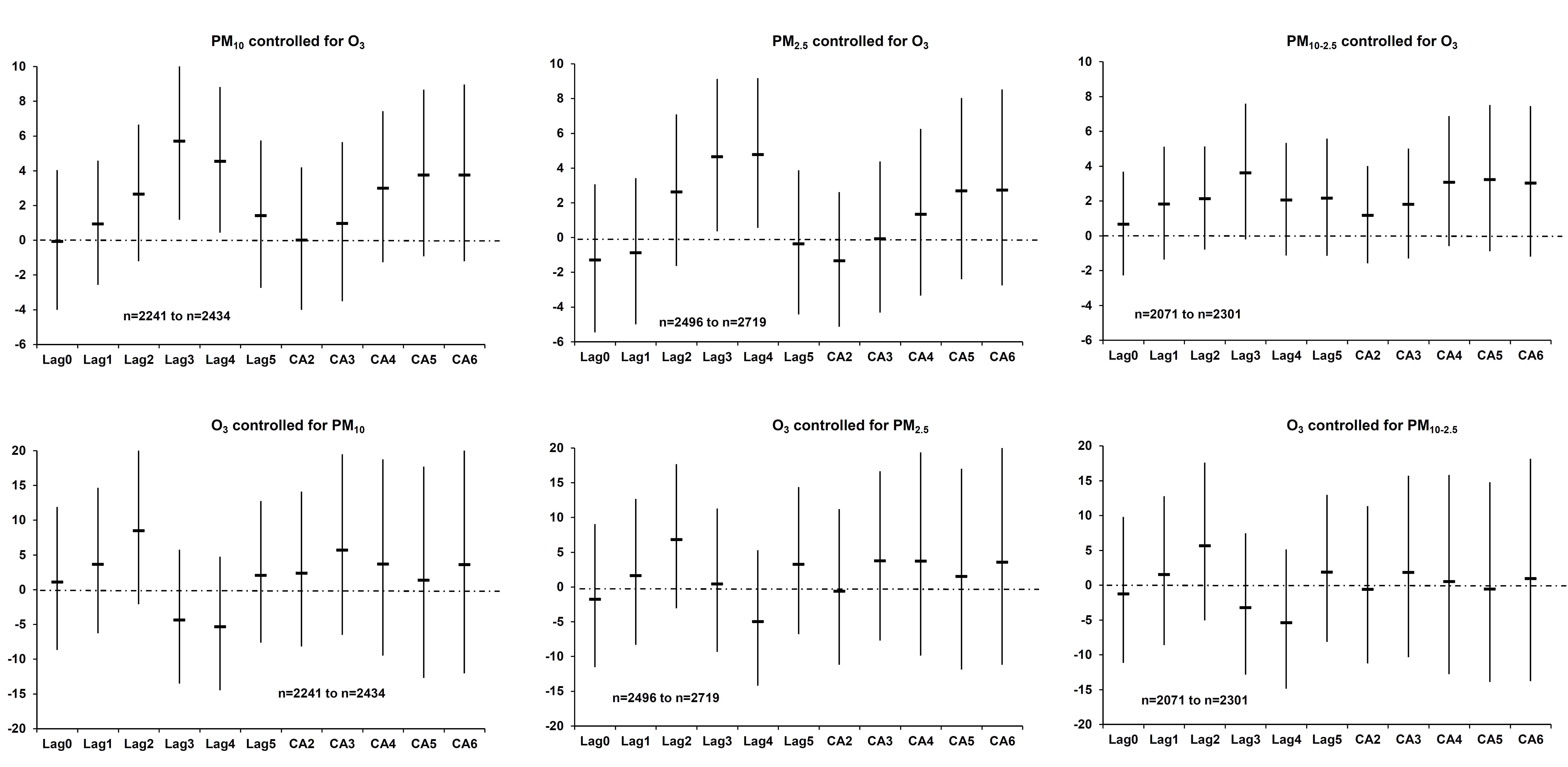


Figure S18. Percentage change (95% CI) in out-of-hospital cardiac arrests in Copenhagen per inter-quartile range increase in *daily* PM_10_, PM_2.5_ and PM_10-2.5_ levels during 1 January 2000−31 December 2010, adjusted for O_3_ (same lag as PM_10_, PM_2.5_ and PM_10-2.5_), public holidays, temperature and relative humidity (same lag as PM_10_, PM_2.5_ and PM_10-2.5_).

**n is the number of OHCA cases used in the models and is less than 4657 due to missing exposure data

Figure S19. Association (expressed as odds ratio) and cumulative association (expressed as odds ratio) between out-of-hospital cardiac arrests and an inter-quartile range increase in the *daily* lag0 to lag5 of PM_2.5_ and PM_10_ in Copenhagen during 1 January 2000−31 December 2010.

The second degree polynomial distributed lag models for were adjusted for the distributed lag0 to lag5 of temperature, relative humidity, public holiday, day of the week and month-year strata.
