# Supplementary material for: Out-of-Hospital Cardiac Arrests and Outdoor Air Pollution Exposure in Copenhagen, Denmark
Source: PLoS One. 2013 Jan 14;8(1):e53684. doi: 10.1371/journal.pone.0053684 (PMC3544842; doi:10.1371/journal.pone.0053684)
Supplement: File S2 — Supplementary tables. (DOC) [file pone.0053684.s002.doc]

**Table S1. Mean difference^a^ between case days and mean of control days for daily exposure levels**

|  | **Lag0** | **Lag1** | **Lag2** | **Lag3** | **Lag4** | **Lag5** |
| --- | --- | --- | --- | --- | --- | --- |
|  | **Difference (95% CI)** | **Difference (95% CI)** | **Difference (95% CI)** | **Difference (95% CI)** | **Difference (95% CI)** | **Difference (95% CI)** |
| **PM_10_ (µg/m^3^)** | -0.04 (-0.39; 0.30) | 0.15 (-0.18; 0.48) | 0.08 (-0.26; 0.42) | 0.30 (-0.03; 0.62) | 0.30 (-0.04; 0.63) | 0.15 (-0.20; 0.49) |
| **PM_2.5_ (µg/m^3^)** | -0.11 (-0.34; 0.11) | -0.08 (-0.31; 0.15) | 0.08 (-0.15; 0.31) | 0.20 (-0.03; 0.42) | 0.25 (0.01; 0.48) | -0.02 (-0.24; 0.21) |
| **PM_10-2.5_ (µg/m^3^)** | 0.08 (-0.21; 0.36) | 0.15 (-0.09; 0.40) | 0.14 (-0.13; 0.40) | 0.20 (-0.04; 0.45) | 0.17 (-0.10; 0.43) | 0.17 (-0.12; 0.46) |
| **PAC (µm^2^/m^3^)** | -5.14 (-10.91; 0.64) | -1.98 (-7.83; 3.87) | -1.71 (-7.64; 4.22) | 1.97 (-4.09; 8.03) | 2.54 (-3.66; 8.75) | 1.35 (-4.50; 7.21) |
| **PVC (µm^3^/m^3^)** | -0.27 (-0.55; 0.02) | -0.14 (-0.42; 0.15) | -0.05 (-0.34; 0.24) | 0.12 (-0.17; 0.42) | 0.11 (-0.20; 0.42) | 0.08 (-0.21; 0.37) |
| **PNC (no./cm^3^)** | -33 (-163; 97) | -32 (-163; 99) | -81 (-215; 52) | -9 (-141; 123) | 51 (-81; 182) | 44 (-90; 179) |
| **NO_x_ (ppb)** | -0.18 (-0.42; 0.06) | 0.02 (-0.22; 0.27) | -0.12 (-0.36; 0.12) | 0.17 (-0.08; 0.41) | 0.10 (-0.14; 0.34) | 0.05 (-0.19; 0.30) |
| **NO_2_ (ppb)** | -0.10 (-0.25; 0.05) | -0.01 (-0.16; 0.14) | -0.10 (-0.25; 0.05) | 0.07 (-0.08; 0.22) | 0.06 (-0.09; 0.21) | 0.00 (-0.15; 0.15) |
| **O_3_ (ppb)** | 0.13 (-0.15; 0.40) | 0.18 (-0.09; 0.46) | 0.18 (-0.09; 0.45) | 0.02 (-0.26; 0.30) | -0.15 (-0.42; 0.12) | 0.05 (-0.22; 0.32) |
| **CO (ppm)** | -0.003 (-0.005; 0.000) | -0.001 (-0.004; 0.002) | -0.002 (-0.005; 0.001) | 0.001 (-0.002; 0.004) | 0.003 (0.000; 0.006) | 0.002 (-0.002; 0.005) |
| **Temperature (°C)** | -0.04 (-0.13; 0.05) | -0.02 (-0.11; 0.07) | -0.04 (-0.14; 0.05) | -0.03 (-0.12; 0.06) | -0.03 (-0.12; 0.06) | -0.06 (-0.14; 0.03) |
| **Relative humidity (%)** | -0.14 (-0.42; 0.14) | -0.20 (-0.49; 0.09) | -0.01 (-0.30; 0.28) | -0.13 (-0.42; 0.15) | -0.07 (-0.36; 0.21) | 0.03 (-0.25; 0.32) |

^a^Differences between case days and control days are calculated by subtracting the average of the level on the associated control days from the case day. The average of these differences for the 4657 OHCA cases is then calculated.

**Table S2. Descriptive statistics for daily PM_2.5_ and PM_10_ levels (lag0) on days that out-of-hospital cardiac arrests occurred in Copenhagen.**

|  |  |  |  |  | **Percentiles** | | |  | **Difference between case days and mean control days**  **(95% CI)**^c^ |
| --- | --- | --- | --- | --- | --- | --- | --- | --- | --- |
|  | **No. days** | **No. days missing data** | **Mean** | **SD** | **25^th^** | **50^th^** | **75^th^** | **IQR** |
| **1 May 2002 − 31 Dec 2010** |  |  |  |  |  |  |  |  |  |
| PM_10_ TEOM (µg/m^3^) | 2159^a^ | 389 | 15.28 | 8.73 | 10.54 | 13.47 | 17.62 | 7.09 | -0.06 (-0.41, 0.28) |
| PM_10_ BA (µg/m^3^) | 2159^a^ | 151 | 24.69 | 12.09 | 16.75 | 22.60 | 29.60 | 12.85 | -0.25 (-0.69, 0.20) |
| **1 Apr 2008 −31 Dec 2010** |  |  |  |  |  |  |  |  |  |
| PM_2.5_ TEOM (µg/m^3^) | 675^b^ | 53 | 9.60 | 5.15 | 6.49 | 8.29 | 10.71 | 4.23 | -0.01 (-0.37, 0.34) |
| PM_2.5_ BA (µg/m^3^) | 675^b^ | 202 | 12.78 | 7.47 | 7.70 | 11.00 | 16.20 | 8.50 | -0.16 (-0.79, 0.47) |

TEOM: Tapered element oscillating microbalance monitor

BA: Beta attenuation monitor

SD: Standard deviation

IQR: Interquartile range

^a^Of the 3167 days during the study period, 3606 OHCAs occurred on 2159 days

^b^Of the 1005 days during the study period, 1117 OHCAs occurred on 675 days

^c^Differences between case days and control days are calculated by subtracting the average of the level on the associated control days from the case day. The average of these differences for the 4657 OHCA cases is then calculated.

**Table S3. Association between air pollutants and out-of-hospital cardiac arrests in Copenhagen, expressed as percentage increase in risk (%) and 95% confidence intervals per inter-quartile increase in daily lag0 to lag4 and 2-day cumulative average (1 May 2002−31 December 2010).**

|  | **PM_10_ TEOM** | | | | | **PM_10_ BA** | | | | |
| --- | --- | --- | --- | --- | --- | --- | --- | --- | --- | --- |
|  | **IQR** | **n**^a^ | **%** | **95% CI** | | **IQR** | **n** | **%** | **95% CI** | |
| **Lag0** | 7 | 2962 | -0.1 | -3.8 | 3.6 | 13 | 3311 | -1.9 | -6.6 | 3.1 |
| **Lag1** | 7 | 2969 | 2.2 | -1.6 | 6.0 | 13 | 3312 | 0.8 | -4.1 | 5.8 |
| **Lag2** | 7 | 2977 | 1.4 | -2.2 | 5.1 | 13 | 3314 | 0.9 | -3.9 | 5.9 |
| **Lag3** | 7 | 2974 | **4.8** | **0.7** | **9.0** | 13 | 3302 | **5.2** | **0.1** | **10.5** |
| **Lag4** | 7 | 2978 | **3.8** | **0.1** | **7.6** | 13 | 3309 | 4.2 | -0.6 | 9.2 |
| **CA2** | 6 | 2928 | 0.7 | -2.9 | 4.5 | 12 | 3212 | -0.8 | -6.0 | 4.6 |

TEOM: Tapered element oscillating microbalance monitor

BA: Beta attenuation monitor

^a^Number of OHCA cases used in the models, which is less than 3606 due to missing exposure data

**Table S4. Association between air pollutants and out-of-hospital cardiac arrests in Copenhagen, expressed as percentage increase in risk (%) and 95% confidence intervals per inter-quartile increase in daily lag0 to lag4 and 2-day cumulative average (1 April 2008−31 December 2010).**

|  | **PM_2.5_ TEOM** | | | | | **PM_2.5_ BA** | | | | |
| --- | --- | --- | --- | --- | --- | --- | --- | --- | --- | --- |
|  | **IQR** | **n**^a^ | **%** | **95% CI** | | **IQR** | **n** | **%** | **95% CI** | |
| **Lag0** | 4 | 1009 | 0.3 | -5.6 | 6.5 | 9 | 779 | -1.3 | -12.0 | 10.7 |
| **Lag1** | 4 | 1022 | 1.6 | -4.2 | 7.7 | 8 | 800 | 3.0 | -6.9 | 14.0 |
| **Lag2** | 4 | 1023 | 6.1 | 0.2 | 12.2 | 9 | 782 | 4.6 | -7.0 | 17.7 |
| **Lag3** | 4 | 1018 | **7.3** | **1.2** | **13.7** | 9 | 806 | 2.3 | -8.3 | 14.3 |
| **Lag4** | 4 | 1013 | **10.4** | **4.6** | **16.6** | 9 | 803 | 10.7 | -0.5 | 23.1 |
| **CA2** | 4 | 1003 | 0.6 | -6.3 | 8.1 | 8 | 715 | 3.6 | -9.3 | 18.3 |

TEOM: Tapered element oscillating microbalance monitor

BA: Beta attenuation monitor

^a^Number of OHCA cases used in the models, which is less than 1117 due to missing exposure data

**Table S5. Spearman correlation coefficients between exposure variables (daily lag0) in Copenhagen (1 May 2002−31 December 2010).**

|  | **PM_10_ BA** | **PM_2.5_ TEOM** | **PM_2.5_ BA** | **PAC** | **PVC** | **PNC** | **NO_x_** | **NO_2_** | **O_3_** | **CO** | **Temp** | **Rel. hum** |
| --- | --- | --- | --- | --- | --- | --- | --- | --- | --- | --- | --- | --- |
| **PM_10_ TEOM** | 0.843 | 0.812 | 0.617 | 0.589 | 0.590 | 0.373 | 0.302 | 0.322 | 0.028^a^ | 0.209 | 0.251 | -0.045^a^ |
|  | 1770 | 1682 | 1405 | 386 | 1025 | 1025 | 1717 | 1717 | 1462 | 1676 | 1755 | 1755 |
| **PM_10_ BA** |  | 0.832 | 0.708 | 0.707 | 0.729 | 0.394 | 0.434 | 0.459 | -0.139 | 0.345 | 0.173 | 0.175 |
|  |  | 1600 | 451 | 1233 | 1233 | 1233 | 1943 | 1943 | 1677 | 1897 | 1972 | 1972 |
| **PM_2.5_ TEOM** |  |  | 0.773 | 0.759 | 0.788 | 0.338 | 0.368 | 0.397 | -0.107 | 0.371 | 0.038^a^ | 0.160 |
|  |  |  | 458 | 1090 | 1090 | 1090 | 1637 | 1637 | 1650 | 1633 | 1665 | 1665 |
| **PM_2.5_ BA** |  |  |  | 0.650 | 0.677 | 0.381 | 0.373 | 0.401 | -0.170 | 0.287 | -0.020^a^ | 0.224 |
|  |  |  |  | 278 | 278 | 278 | 474 | 474 | 474 | 457 | 463 | 463 |
| **PAC** |  |  |  |  | 0.975 | 0.691 | 0.491 | 0.530 | -0.200 | 0.476 | -0.048^a^ | 0.240 |
|  |  |  |  |  | 1267 | 1267 | 1252 | 1252 | 1111 | 1242 | 1256 | 1256 |
| **PVC** |  |  |  |  |  | 0.557 | 0.462 | 0.501 | -0.261 | 0.491 | -0.095 | 0.344 |
|  |  |  |  |  |  | 1267 | 1252 | 1252 | 1111 | 1242 | 1256 | 1256 |
| **PNC** |  |  |  |  |  |  | 0.453 | 0.474 | -0.015^a^ | 0.323 | 0.031^a^ | -0.057^a^ |
|  |  |  |  |  |  |  | 1252 | 1252 | 1111 | 1242 | 1256 | 1256 |
| **NO_x_** |  |  |  |  |  |  |  | 0.983 | -0.548 | 0.539 | -0.108 | 0.247 |
|  |  |  |  |  |  |  |  | 2070 | 1733 | 1987 | 2045 | 2045 |
| **NO_2_** |  |  |  |  |  |  |  |  | -0.520 | 0.557 | -0.123 | 0.251 |
|  |  |  |  |  |  |  |  |  | 1733 | 1987 | 2045 | 2045 |
| **O_3_** |  |  |  |  |  |  |  |  |  | -0.476 | 0.362 | -0.654 |
|  |  |  |  |  |  |  |  |  |  | 1695 | 1754 | 1754 |
| **CO** |  |  |  |  |  |  |  |  |  |  | -0.503 | 0.380 |
|  |  |  |  |  |  |  |  |  |  |  | 2003 | 2003 |
| **Temp** |  |  |  |  |  |  |  |  |  |  |  | -0.375 |
|  |  |  |  |  |  |  |  |  |  |  |  | 2101 |

TEOM: Tapered element oscillating microbalance monitor

BA: Beta attenuation monitor

Top value is the Spearman correlation coefficient and the bottom value is the sample size

^a^p-value > 0.05, otherwise p-value < 0.01

**Table S6. Spearman correlation coefficients between exposure variables (daily lag0) in Copenhagen (1 April 2008−31 December 2010).**

|  | **PM_10_ BA** | **PM_2.5_ TEOM** | **PM_2.5_ BA** | **PAC** | **PVC** | **PNC** | **NO_x_** | **NO_2_** | **O_3_** | **CO** | **Temp** | **Rel. hum** |
| --- | --- | --- | --- | --- | --- | --- | --- | --- | --- | --- | --- | --- |
| **PM_10_ TEOM** | 0.834 | 0.839 | 0.617 | 0.583 | 0.573 | 0.410 | 0.223 | 0.249 | 0.137 | 0.006^a^ | 0.339 | -0.163 |
|  | 503 | 520 | 386 | 252 | 252 | 252 | 496 | 496 | 530 | 506 | 526 | 526 |
| **PM_10_ BA** |  | 0.830 | 0.705 | 0.688 | 0.686 | 0.440 | 0.397 | 0.425 | -0.030^a^ | 0.150 | 0.214 | 0.067^a^ |
|  |  | 580 | 447 | 317 | 317 | 317 | 570 | 570 | 601 | 573 | 586 | 586 |
| **PM_2.5_ TEOM** |  |  | 0.771 | 0.710 | 0.714 | 0.404 | 0.344 | 0.375 | -0.039^a^ | 0.266 | 0.057^a^ | 0.127 |
|  |  |  | 454 | 323 | 323 | 323 | 587 | 587 | 620 | 600 | 606 | 606 |
| **PM_2.5_ BA** |  |  |  | 0.653 | 0.679 | 0.381 | 0.372 | 0.399 | -0.175 | 0.284 | -0.020^a^ | 0.227 |
|  |  |  |  | 274 | 274 | 274 | 470 | 470 | 470 | 453 | 459 | 459 |
| **PAC** |  |  |  |  | 0.974 | 0.747 | 0.487 | 0.513 | -0.305 | 0.345 | -0.048^a^ | 0.310 |
|  |  |  |  |  | 324 | 324 | 324 | 324 | 324 | 322 | 314 | 314 |
| **PVC** |  |  |  |  |  | 0.626 | 0.443 | 0.475 | -0.348 | 0.352 | -0.109^a^ | 0.395 |
|  |  |  |  |  |  | 324 | 324 | 324 | 324 | 322 | 314 | 314 |
| **PNC** |  |  |  |  |  |  | 0.509 | 0.515 | -0.123 | 0.246 | 0.133 | 0.003^a^ |
|  |  |  |  |  |  |  | 324 | 324 | 324 | 322 | 314 | 314 |
| **NO_x_** |  |  |  |  |  |  |  | 0.988 | -0.491 | 0.377 | -0.132 | 0.196 |
|  |  |  |  |  |  |  |  | 618 | 618 | 596 | 602 | 602 |
| **NO_2_** |  |  |  |  |  |  |  |  | -0.457 | 0.386 | -0.144 | 0.203 |
|  |  |  |  |  |  |  |  |  | 618 | 596 | 602 | 602 |
| **O_3_** |  |  |  |  |  |  |  |  |  | -0.384 | 0.441 | -0.677 |
|  |  |  |  |  |  |  |  |  |  | 624 | 636 | 636 |
| **CO** |  |  |  |  |  |  |  |  |  |  | -0.520 | 0.321 |
|  |  |  |  |  |  |  |  |  |  |  | 609 | 609 |
| **Temp** |  |  |  |  |  |  |  |  |  |  |  | -0.488 |
|  |  |  |  |  |  |  |  |  |  |  |  | 636 |

TEOM: Tapered element oscillating microbalance monitor

BA: Beta attenuation monitor

Top value is the Spearman correlation coefficient and the bottom value is the sample size

^a^p-value > 0.05, otherwise p-value < 0.05

**Table S7. Association between PM_2.5_ and PM_10_ (also adjusted for O_3_) and out-of-hospital cardiac arrests in Copenhagen, expressed as percentage increase in risk (%) and 95% confidence intervals per inter-quartile increase in daily lag0 to lag5 and 2-day, 4-day and 6-day cumulative average (1 January 2000−31 December 2010).**

|  | **PM_2.5_ adjusted for O_3_** | | | | | **O_3_ adjusted for PM_2.5_** | | | | |
| --- | --- | --- | --- | --- | --- | --- | --- | --- | --- | --- |
|  | **IQR** | **n^a^** | **%** | **95% CI** | | **IQR** | **n^a^** | **%** | **95% CI** | |
| **Lag0** | 5 | 2692 | -1.3 | -5.5 | 3.1 | 14 | 2692 | -1.8 | -11.5 | 9.0 |
| **Lag1** | 5 | 2706 | -0.9 | -5.0 | 3.4 | 14 | 2706 | 1.6 | -8.3 | 12.7 |
| **Lag2** | 5 | 2719 | 2.6 | -1.6 | 7.1 | 13 | 2719 | 6.8 | -3.0 | 17.7 |
| **Lag3** | 5 | 2717 | **4.7** | **0.4** | **9.1** | 14 | 2717 | 0.5 | -9.3 | 11.3 |
| **Lag4** | 5 | 2718 | **4.8** | **0.6** | **9.2** | 14 | 2718 | -5.0 | -14.2 | 5.3 |
| **CA2** | 4 | 2659 | -1.3 | -5.1 | 2.6 | 13 | 2659 | -0.6 | -11.2 | 11.2 |
|  | **PM_10_ adjusted for O_3_** | | | | | **O_3_ adjusted for PM_10_** | | | | |
|  | **IQR** | **n^a^** | **%** | **95% CI** | | **IQR** | **n^a^** | **%** | **95% CI** | |
| **Lag0** | 7 | 2413 | -0.1 | -4.0 | 4.0 | 13 | 2413 | 1.1 | -8.7 | 11.9 |
| **Lag1** | 6 | 2417 | 0.9 | -2.6 | 4.6 | 13 | 2417 | 3.7 | -6.3 | 14.7 |
| **Lag2** | 7 | 2423 | 2.7 | -1.2 | 6.7 | 13 | 2423 | 8.5 | -2.1 | 20.2 |
| **Lag3** | 7 | 2422 | **5.7** | **1.2** | **10.4** | 13 | 2422 | -4.4 | -13.5 | 5.7 |
| **Lag4** | 7 | 2425 | **4.5** | **0.4** | **8.8** | 13 | 2425 | -5.3 | -14.5 | 4.8 |
| **CA2** | 6 | 2380 | 0.0 | -4.0 | 4.2 | 12 | 2380 | 2.4 | -8.2 | 14.1 |
|  | **PM_10-2.5_ adjusted for O_3_** | | | | | **O_3_ adjusted for PM_10-2.5_** | | | | |
|  | **IQR** | **n^a^** | **%** | **95% CI** | | **IQR** | **n^a^** | **%** | **95% CI** | |
| **Lag0** | 4 | 2270 | 0.7 | -2.3 | 3.7 | 13 | 2270 | -1.2 | -11.2 | 9.8 |
| **Lag1** | 4 | 2288 | 1.8 | -1.4 | 5.1 | 13 | 2288 | 1.5 | -8.6 | 12.8 |
| **Lag2** | 4 | 2296 | 2.1 | -0.8 | 5.1 | 13 | 2296 | 5.7 | -5.0 | 17.6 |
| **Lag3** | 4 | 2295 | 3.6 | -0.2 | 7.6 | 13 | 2295 | -3.2 | -12.8 | 7.5 |
| **Lag4** | 4 | 2295 | 2.1 | -1.1 | 5.3 | 13 | 2295 | -5.4 | -14.8 | 5.2 |
| **CA2** | 3 | 2235 | 1.2 | -1.6 | 4.0 | 12 | 2235 | -0.6 | -11.2 | 11.3 |

^a^Number of OHCA cases used in the models, which is less than 4657 due to missing exposure data
